# Abdominal Aortic Calcification and Cardiovascular Outcomes in Chronic Kidney Disease: Findings from KNOW-CKD Study

**DOI:** 10.3390/jcm11051157

**Published:** 2022-02-22

**Authors:** Sang Heon Suh, Tae Ryom Oh, Hong Sang Choi, Chang Seong Kim, Eun Hui Bae, Kook-Hwan Oh, Joongyub Lee, Yun Kyu Oh, Ji Yong Jung, Seong Kwon Ma, Soo Wan Kim, on behalf of the Korean Cohort Study for Outcomes in Patients with Chronic Kidney Disease (KNOW-CKD) Investigators

**Affiliations:** 1 Department of Internal Medicine, Chonnam National University Medical School and Chonnam National University Hospital, Gwangju 61469, Korea; medssh1984@gmail.com (S.H.S.); tryeomoh@hanmail.net (T.R.O.); hongsang38@hanmail.net (H.S.C.); laminion@hanmail.net (C.S.K.); baedak76@gmail.com (E.H.B.); 2 Department of Internal Medicine, Seoul National University Hospital, Seoul 03080, Korea; ohchris@hanmail.net; 3 Department of Prevention and Management, School of Medicine, Inha University, Incheon 22212, Korea; tp240@naver.com; 4 Department of Internal Medicine, Seoul National University, Seoul 08826, Korea; yoonkyuoh@gmail.com; 5 Division of Nephrology, Department of Internal Medicine, Gachon University of Gil Medical Center, Incheon 21565, Korea; jyjung@gachon.ac.kr

**Keywords:** chronic kidney disease, abdominal aortic calcification, cardiovascular events, all-cause death

## Abstract

To investigate the association between abdominal aortic calcification score (AACS) assessed by plain radiograph of the lateral abdomen and the risk of cardiovascular (CV) events in patients with pre-dialysis chronic kidney disease (CKD), a total of 2090 pre-dialysis CKD patients from the Korean Cohort Study for Outcome in Patients with Chronic Kidney Disease (KNOW-CKD) were categorized by AACS into 0, 1–2, 3–4, 5–6, and ≥7. The primary outcome of the study was the composite CV events, defined as a composite of non-fatal CV events and all-cause death. The risk of composite CV events was significantly higher in the subjects with AACS ≥ 7 (adjusted hazard ratio (HR) 1.888, 95% confidence interval (CI) 1.219 to 2.923), compared to that of the subjects with AACS 0. The risks of fatal and non-fatal CV events (adjusted HR 1.052, 95% CI 1.030 to 1.073) and all-cause death (adjusted HR 1.949, 95% CI 1.073 to 3.539) were also significantly higher in the subjects with AACS ≥ 7. In conclusion, AACS assessed by plain radiograph is independently associated with adverse CV outcomes in patients with pre-dialysis CKD. A simple radiographic examination of the lateral abdomen may help CV risk stratification in this population.

## 1. Introduction

Cardiovascular disease (CVD) is a leading cause of mortality and morbidity in patients with chronic kidney disease (CKD) [1,2]. Accelerated atherosclerosis and vascular calcification during the progression of CKD impose more frequent cardiovascular (CV) events in this population [3,4,5]. In particular, coronary artery disease (CAD) and heart failure comprise a major CVD burden in patients with CKD [6,7]. Accordingly, the risk stratification for CAD in patients with CKD is an issue of clinical importance. The assessment of coronary artery calcification by cardiac computed tomography (CT) scan sensitively detects CAD, and has been validated for the prediction of future CV event risk [8,9,10,11]. Yet, due to the limitations of thoracic radiation and the cost of cardiac CT [12], alternative modalities have been examined to estimate the CVD burden.

Abdominal aortic calcification (AAC) is common and has been associated with an increased risk of CV events in patients with CKD [13,14,15]. AAC assessed by non-contrast CT scan proved its correlation with coronary artery calcium score (CACS) [16]. AAC derived dual–energy X-ray absorptiometry also predicts the risk of future CV events both in the general population [17] and in patients with CKD [18]. A plain radiograph of the lateral abdomen also provides a simple, low-cost means to evaluate AAC [19], and has been associated with an increased risk of CV events and all-cause death, especially in patients with end-stage renal disease [13,20,21], while its predictive value has been only limitedly validated in patients with pre-dialysis CKD [22].

Taking advantage of a cohort of 2090 participants with pre-dialysis CKD, we aimed to investigate the association between AAC score (AACS) assessed by plain radiograph of the lateral abdomen and the risk of CV events. We also evaluated the association of AACS with the risk of all-cause death in this population. In addition, we conducted a series of subgroup analyses to determine whether the association between AACS and the risk of CV events is modified by clinical contexts.

## 2. Materials and Methods

### 2.1. Study Design

**Figure 1:**
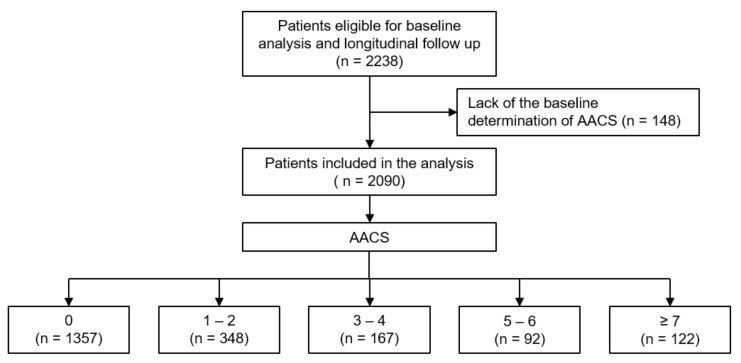
Flow diagram of the study participants. Abbreviations: AACS, abdominal aortic calcification score.

The Korean Cohort Study for Outcomes in Patients with Chronic Kidney Disease (KNOW-CKD) is a nationwide prospective cohort study involving nine tertiary-care general hospitals in Korea (NCT01630486 at http://www.clinicaltrials.gov, accessed on 12 January 2022) [23]. Korean patients with CKD from stage 1 to pre-dialysis stage 5, who voluntarily provided informed consent, were enrolled from 2011 through 2016. The study was conducted in accordance with the principles of the Declaration of Helsinki. The study protocol was approved by the institutional review boards of the participating centers, including at Seoul National University Hospital, Yonsei University Severance Hospital, Kangbuk Samsung Medical Center, Seoul St. Mary’s Hospital, Gil Hospital, Eulji General Hospital, Chonnam National University Hospital, and Busan Paik Hospital. All participants had been under close observation, and participants who experienced study outcomes were reported by each participating center. Among 2238 individuals who were longitudinally followed up, excluding those lacking the baseline determination of AACS, a total of 2090 subjects were finally included for the analyses (Figure 1). The study observation period ended on 31 March 2020. The median follow-up duration was 5.9 years.

### 2.2. Data Collection from Participants

Demographic information was collected from all eligible participants, including age, gender, comorbid conditions, primary renal disease, smoking history, and medication history (angiotensin-converting enzyme inhibitor/angiotensin II receptor blockers (ACEi/ARBs), diuretics, number of anti-HTN drugs, statins). Trained staff members measured the height and weight of the study participants. Body mass index (BMI) was calculated as weight divided by the height squared. Systolic and diastolic blood pressures (SBP and DBP) were measured by an electronic sphygmomanometer after seated rest for 5 min. Venous samples were collected following overnight fasting, to determine hemoglobin, albumin, total cholesterol, low density lipoprotein cholesterol, high density lipoprotein cholesterol (HDL-C), triglyceride (TG), fasting glucose, high-sensitivity C-reactive protein (hs-CRP), 25-hydroxyvitamin D (25(OH) vitamin D) and creatinine (Cr) levels at the baseline. eGFR was calculated by Chronic Kidney Disease Epidemiology Collaboration equation [24]. CKD stages were determined by the Kidney Disease Improving Global Outcomes guidelines [25]. Urine albumin-to-creatinine ratio (ACR) was measured once in random, preferably second-voided, spot urine samples.

### 2.3. Determination of AACS

AACS was determined as previously described [19]. Briefly, calcific deposits in the abdominal aorta adjacent to each lumbar vertebra were assessed separately for the posterior and anterior wall of the aorta. Lesions were graded as follows: 0, no aortic calcific deposits; 1, small scattered calcific deposits filling less than one third of the longitudinal wall of the aorta; 2, one third or more, but less than two thirds of the longitudinal wall of the aorta calcified; 3, two thirds or more of the longitudinal wall of the aorta calcified. The scores of individual aortic segments both for the posterior and anterior wall were summed to yield the antero–posterior severity score (0–24), which was defined as AACS in the present study. The study participants were categorized by AACS at the baseline as follows, which was a priori designed based on the consensus of the participating researchers of KNOW-CKD at the beginning of the cohort study (Figure 1): 0, 1–2, 3–4, 5–6, ≥7.

### 2.4. Echocardiographic Data Collection

Complete two-dimensional M-mode and Doppler studies were performed via standard approaches by cardiologists at the participating hospitals who were blinded to the clinical data. M-mode examination was performed according to American Society of Echocardiography guidelines [26]. The recorded echocardiographic data were the ratio of the early transmitral blood flow velocity to early diastolic velocity of the mitral annulus (E/e’), left ventricular ejection fraction (LVEF), regional wall motion abnormality, valve calcification, left ventricular (LV) posterior wall thickness, inter-ventricular septum thickness, LV end diastolic diameter, and LV end systolic diameter. E/e’ > 14 was defined as high [27,28]. LV mass was determined using the Devereux formula [26]. LV mass index was calculated by normalizing LV mass to height^2^ (g/m^2^).

### 2.5. Study Outcomes

The primary outcome of the present study was the composite CV events, defined as a composite of non-fatal CV events and all-cause death. The secondary outcomes were fatal and non-fatal CV events and all-cause death. CV events included any coronary artery event (unstable angina, myocardial infarction, or coronary intervention/surgery), hospitalization for heart failure, ischemic or hemorrhagic stroke, or symptomatic arrhythmia.

### 2.6. Statistical Analysis

Continuous variables were expressed as mean ± standard deviation or median (interquartile range). Categorical variables were expressed as number of participants and percentage. Normality of distribution was ascertained by the Kolmogorov–Smirnov test. To compare the baseline characteristics by AACS, one-way analysis of variance and χ^2^ test were used for the continuous and categorical variates, respectively. In the primary analysis, the participants with any missing data were excluded for further analyses. To address the association between AACS and study outcomes, Cox proportional hazard regression models were analyzed. Patients lost to follow-up were censored at the date of the last visit. Models were constructed after adjusting for the following variables. Model 1 represents crude hazard ratios (HRs). Model 2 was adjusted for age, sex, Charlson comorbidity index, current smoking status, medication (ACEi/ARBs, diuretics, number of antihypertensive drugs, statins), BMI, and SBP. Model 3 was further adjusted for hemoglobin, albumin, fasting glucose, HDL-C, TG, 25(OH) vitamin D, hs-CRP, eGFR, and spot urine ACR. Model 4 was additionally adjusted for LVEF and categorized E/e’ at the baseline. The results of the Cox proportional hazard models were presented as HRs and 95% confidence intervals (CIs). Cumulative incidences of composite CV events, fatal and non-fatal CV events, and all-cause death were estimated using Kaplan–Meier analyses, and were compared using log-rank test. To validate our findings, we performed sensitivity analyses. First, we excluded the subjects with eGFR < 15 mL/min./1.73 m^2^, because the subjects with eGFR < 15 mL/min./1.73 m^2^ are relatively small in number, and may exaggerate the association between AACS and study outcomes due to far advanced CKD. Second, we excluded the subjects with eGFR ≥ 90 mL/min./1.73 m^2^, because the subjects with eGFR ≥ 90 mL/min./1.73 m^2^ are considered close to normal kidney function, and may not represent CKD population well. Lastly, we replaced the missing values in the primary analyses by a multiple imputation, and further conducted Cox regression analyses. To examine whether the association of AACS with the study outcomes is modified by certain clinical contexts, we conducted pre-specified subgroup analyses. Subgroups were defined by age (<60 versus vs. ≥60 years), sex (male vs. female), BMI (<23 vs. ≥23 kg/m^2^), eGFR (<45 vs. ≥45 mL/min/1.73 m^2^), and spot urine ACR (<300 vs. ≥300 mg/gCr). Two-sided *p* values < 0.05 were considered statistically significant. Statistical analysis was performed using SPSS for Windows version 22.0 (IBM Corp., Armonk, NY, USA) and R (version 4.1.1; R project for Statistical Computing, Vienna, Austria).

## 3. Results

### 3.1. Baseline Characteristics

The baseline characteristics of the study participants were described by AACS (Table 1). The mean follow-up duration was shortest in the subjects with AACS ≥ 7, while the mean age and the proportion of male sex were highest in the subjects with AACS ≥ 7. The proportion of Charlson comorbidity index ≥ 4 and the frequency of DM history increased as AACS increased. Diuretic use, medication of no less than three anti-hypertensives, and statin medication were most prevalent in the subjects with AACS ≥ 7. BMI and SBP also increased as AACS increased. Hemoglobin, total cholesterol, and HDL-C levels were lowest in the subjects with AACS ≥ 7, whereas TG, fasting glucose, hs-CRP, and spot urine ACR levels were highest in the subjects with AACS ≥ 7. Albumin levels were lowest in the subjects with AACS 5–6. eGFR significantly decreased as AACS increased. The echocardiographic findings (Supplementary Table S1) revealed that, although LVEF was not significantly different across the groups, E/e’, LV mass index, LV posterior wall thickness, and large inter-ventricular septum thickness positively correlated with AACS. The proportion of RWMA and valve calcification was highest in the subjects with AACS 5–6. LV end diastolic diameter, and LV end systolic diameter were not significantly different among the groups.

### 3.2. Association of AACS with the Risk of Composite CV Events in CKD

**Figure 2:**
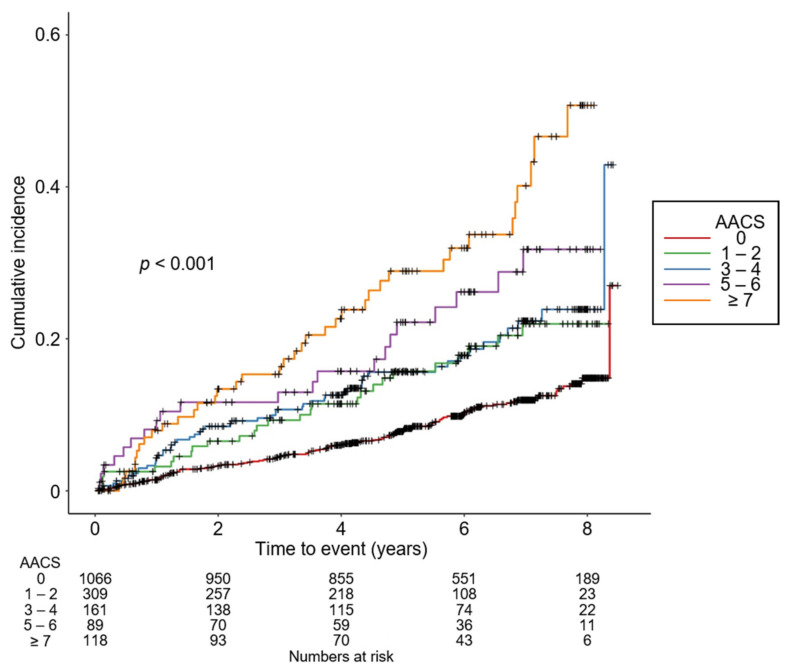
Kaplan–Meier curve for cumulative incidence of composite CV events by AACS. *p* value by log-rank test. Abbreviations: AACS, abdominal aortic calcification score.

To compare the cumulative incidences of composite CV events (Figure 2), fatal and non-fatal CV events (Supplementary Figure S1), and all-cause death (Supplementary Figure S2), Kaplan–Meier analyses were conducted. The risks of composite CV events, fatal and non-fatal CV events, and all-cause death were significantly differed by AACS (all *p* < 0.001, by log-rank test). To define the independent association of AACS with the study outcomes, Cox regression models were analyzed. The risk of composite CV events was significantly higher in the subjects with AACS ≥ 7 (adjusted HR 1.888, 95% CI 1.219 to 2.923) compared to that of the subjects with AACS 0 (Table 2). The risks of fatal and non-fatal CV events (adjusted HR 1.052, 95% CI 1.030 to 1.073) and all-cause death (adjusted HR 1.949, 95% CI 1.073 to 3.539) were significantly higher in the subjects with AACS ≥ 7 compared to those of the subjects with AACS 0 (Table 3).

### 3.3. Sensitivity Analysis

After excluding the subjects at CKD stage 5, who are relatively small in number and may exaggerate the association between AACS and study outcomes due to far advanced CKD, the risk of composite CV events was still significantly higher in the subjects with AACS ≥ 7 (adjusted HR 1.837, 95% CI 1.155 to 2.921) compared to that of the subjects with AACS 0 (Supplementary Table S2). After excluding the subjects at CKD stage 1, who are considered close to normal kidney function and may not represent the CKD population well, the association between AACS with the risk of composite CV events was robustly significant (adjusted HR 1.872, 95% CI 1.201 to 2.919) (Supplementary Table S3). Lastly, after replacing the missing values by multiple imputation, the risk of composite CV events remained significantly higher in the subjects with AACS ≥ 7 (adjusted HR 1.604, 95% CI 1.062 to 2.422) compared to that of the subjects with AACS 0 (Supplementary Table S4).

### 3.4. Subgroup Analysis

Subgroup analyses revealed that the association of AACS with the risk of composite CV events is not modified by age, sex, BMI, eGFR, or albuminuria (Table 4).

## 4. Discussion

In the present study, we demonstrated that AACS assessed by plain radiograph is independently associated with adverse CV outcomes as well as all-cause death in patients with pre-dialysis CKD. We also revealed that the associations were not modified by various clinical contexts, such as age, sex, body mass index, estimated glomerular filtration rate, and albuminuria.

It has long been reported that, based on the autopsy of more than 600 adults, there is a significant association between the degree of AAC and the presence of coronary artery calcification [29]. Previous studies demonstrated that the severity of AAC assessed by plain lateral abdomen radiograph is closely related to CV morbidity and mortality in the general population [30,31]. While the association has been validated in patients with end-stage renal disease [13,20,21], there is limited data supporting the association in patients with pre-dialysis CKD [22]. In this regard, our study presents compelling evidence that a simple radiographic examination of the lateral abdomen may help CV risk stratification in patients with pre-dialysis CKD.

The predictability of AACS in future CV events seems primarily attributed to its correlation with coronary artery calcification. Previous studies reported that, among the patients on chronic hemodialysis, a high AACS on plain radiograph is independently associated with severe CACS on cardiac CT [12,32,33]. Although there is no direct evidence that AACS assessed by plain radiograph correlates with CACS in patients with non-dialysis CKD, abdominal CT-assessed AAC’s correlation with CACS is proven [16]. Therefore, it seems reasonable that AACS by plain radiograph predicts the risk of CV events via mirroring the underlying CAD in patients with pre-dialysis CKD.

There are number of limitations to be acknowledged in the current study. First, we cannot determine the causal relation between AACS and the study outcomes, because of the observational nature of the current study. Second, albeit AAC can be assessed by various methods, such as non-contrast CT and dual-energy X-ray absorptiometry, we did not determine which method is the best predictor of future CV event risk. Third, some of data in the present study, such as the echocardiographic measurements, were obtained from individual participating centers and were not centralized, whereas the multicenter nature of the current study is a strength. Fourth, as this cohort study only enrolled ethnic Koreans, caution is required in extrapolating the data to other populations.

## 5. Conclusions

In conclusion, we reported that AACS assessed by plain radiograph is independently associated with adverse CV outcomes and all-cause death in patients with pre-dialysis CKD. It is expected that a simple radiographic examination of the lateral abdomen may help CV risk stratification in this population.

## Figures and Tables

**Table 1:** Baseline characteristics of study participants by AACS.

	AACS					*p* Value
	0	1–2	3–4	5–6	≥7	
Age (year)	50.2 ± 12.0	57.0 ± 10.3	60.1 ± 9.2	63.2 ± 9.2	65.0 ± 7.0	<0.001
Male	798 (58.8)	226 (64.9)	111 (66.5)	61 (66.3)	81 (66.4)	<0.001
Charlson comorbidity index						0.046
0–3	15114 (81.9)	211 (60.6)	95 (56.9)	42 (45.7)	32 (26.2)	<0.001
4–5	238 (17.5)	126 (36.2)	67 (40.1)	45 (48.9)	86 (70.5)	
6	9 (0.7)	11 (3.2)	5 (3.0)	5 (5.4)	4 (3.3)	
Primary renal disease						
DM	210 (15.5)	121 (34.8)	70 (42.2)	43 (47.3)	79 (64.8)	<0.001
HTN	261 (19.2)	76 (21.8)	36 (21.7)	23 (25.3)	21 (17.2)	
GN	521 (38.4)	79 (22.7)	28 (16.9)	15 (16.5)	11 (9.0)	
TID	11 (0.8)	1 (0.3)	1 (0.6)	0 (0.0)	1 (0.8)	
PKD	275 (20.3)	48 (13.8)	14 (8.4)	1 (1.1)	2 (1.6)	
Others	79 (5.8)	23 (6.6)	17 (10.2)	9 (9.9)	8 (6.6)	
Current smoking status	216 (15.9)	48 (13.8)	36 (21.6)	15 (16.3)	14 (11.5)	
Medication						0.138
ACEi/ARBs	1160 (85.5)	299 (85.9)	135 (80.8)	82 (89.1)	108 (88.5)	
Diuretics	355 (26.2)	119 (34.2)	63 (37.7)	45 (48.9)	71 (58.2)	0.302
Number of anti-HTN drugs ≥ 3	317 (23.4)	113 (32.5)	72 (43.1)	35 (38.0)	64 (52.5)	<0.001
Statins	617 (45.5)	205 (58.9)	117 (70.1)	49 (53.3)	91 (74.6)	<0.001
BMI (kg/m^2^)	24.4 ± 3.5	24.8 ± 3.3	24.6 ± 2.9	25.3 ± 3.6	25.3 ± 3.2	<0.001
SBP (mmHg)	126.3 ± 15.5	129.1 ± 16.5	131.1 ± 17.5	133.0 ± 17.6	133.0 ± 18.7	0.013
DBP (mmHg)	77.2 ± 10.7	77.8 ± 11.6	77.2 ± 11.7	76.4 ± 11.9	73.0 ± 12.6	<0.001
Laboratory findings						0.007
Hemoglobin (g/dL)	13.1 ± 2.0	12.7 ± 2.1	12.4 ± 2.0	12.3 ± 1.9	11.8 ± 1.9	
Albumin (g/dL)	4.2 ± 0.4	4.1 ± 0.4	4.1 ± 0.4	4.1 ± 0.5	4.1 ± 0.5	0.002
Total cholesterol (mg/dL)	176.9 ± 37.9	171.9 ± 41.2	167.0 ± 37.9	165.3 ± 43.7	164.1 ± 40.3	<0.001
HDL-C (mg/dL)	51.0 ± 15.5	47.5 ± 15.0	45.5 ± 14.6	44.4 ± 14.6	44.2 ± 15.1	<0.001
LDL-C (mg/dL)	99.3 ± 31.4	94.0 ± 32.9	90.6 ± 31.3	90.4 ± 28.1	89.7 ± 30.4	<0.001
TG (mg/dL)	153.7 ± 96.9	160.8 ± 106.0	170.2 ± 96.1	165.9 ± 104.8	172.7 ± 108.2	0.093
Fasting glucose (mg/dL)	106.4 ± 36.4	117.8 ± 46.9	118.3 ± 43.9	118.9 ± 37.9	128.0 ± 46.2	<0.001
25(OH) vitamin D (ng/dL)	17.8 ± 8.0	18.3 ± 7.3	17.1 ± 6.7	16.6 ± 7.5	18.1 ± 8.2	0.219
hsCRP (mg/dL)	0.6 (0.2, 1.6)	0.6 (0.3, 1.7)	0.7 (0.3, 1.7)	0.8 (0.5, 2.0)	1.1 (0.5, 2.0)	0.064
Spot urine ACR (mg/g Cr)	301.8 (60.4, 907.1)	403.2 (97.9, 1435.6)	344.0 (72.2, 1484.8)	500.2 (147.0, 1195.0)	633.8 (132.7, 1617.7)	<0.001
eGFR (mL/min./1.73 m^2^)	55.4 ± 32.1	46.1 ± 26.7	40.1 ± 22.0	38.8 ± 19.1	36.4 ± 21.2	<0.001
CKD stages						<0.001
Stage 1	291 (21.4)	39 (11.2)	6 (3.6)	3 (3.3)	4 (3.3)	
Stage 2	288 (21.2)	57 (16.4)	31 (18.6)	10 (10.9)	13 (10.7)	
Stage 3a	222 (16.4)	65 (18.7)	23 (13.8)	20 (21.7)	14 (11.5)	
Stage 3b	241 (17.8)	85 (24.4)	46 (27.5)	27 (29.3)	33 (27.0)	
Stage 4	242 (17.8)	77 (22.1)	48 (28.7)	26 (28.3)	50 (41.0)	
Stage 5	73 (5.4)	25 (7.2)	13 (7.8)	6 (6.5)	8 (6.6)	

Values for categorical variables are given as number (percentage); values for continuous variables, as mean ± standard deviation or median (interquartile range). Abbreviations: ACEi, angiotensin converting enzyme inhibitor; AACS, abdominal aortic calcification score; ACR, albumin-to-creatinine ratio; ARB, angiotensin receptor blocker; BMI, body mass index; CKD, chronic kidney disease; Cr, creatinine; DBP, diastolic blood pressure; DM, diabetes mellitus; eGFR, estimated glomerular filtration rate; GN, glomerulonephritis; HDL-C, high density lipoprotein cholesterol; hsCRP, high-sensitivity C-reactive protein; HTN, hypertension; LDC-C, low density lipoprotein cholesterol; PKD, polycystic kidney disease; SBP, systolic blood pressure; TG, triglyceride; TID, tubulointerstitial disease.

**Table 2:** Cox regression analysis of AACS for the primary outcome (non-fatal CV events and all-cause death).

	AACS	Events, *n* (%)	Model 1		Model 2		Model 3		Model 4	
			HR (95% CIs)	*p* Value	HR (95% CIs)	*p* Value	HR (95% CIs)	*p* Value	HR (95% CIs)	*p* Value
Composite CV events	0	119 (8.8)	Reference		Reference		Reference		Reference	
	1–2	54 (15.5)	1.921 (1.344, 2.745)	<0.001	1.280 (0.915, 1.789)	0.149	1.144 (0.797, 1.643)	0.464	1.169 (0.806, 1.696)	0.411
	3–4	27 (16.2)	1.696 (1.053, 2.734)	0.030	0.961 (0.619, 1.493)	0.860	0.834 (0.514, 1.353)	0.462	0.859 (0.524, 1.408)	0.546
	5–6	22 (23.9)	3.134 (1.924, 5.107)	<0.001	1.175 (0.722, 1.910)	0.516	1.062 (0.632, 1.783)	0.821	1.155 (0.680, 1.962)	0.593
	≥7	40 (32.8)	4.867 (3.320, 7.135)	<0.001	1.631 (1.095, 2.430)	0.0161	1.718 (1.129, 2.614)	0.012	1.888 (1.219, 2.923)	0.004

Model 1, unadjusted model. Model 2, model 1 + adjusted for age, sex, Charlson comorbidity index, primary renal disease, current smoking status, medication (ACEi/ARBs, diuretics, number of anti-HTN drugs, statins), BMI, and SBP. Model 3, model 2 + adjusted for hemoglobin, albumin, fasting glucose, HDL-C, TG, 25(OH) vitamin D, hs-CRP, GFR and spot urine ACR. Model 4, model 3 + adjusted for LVEF and categorized E/e’ at the baseline. Abbreviations: AACS, abdominal aortic calcification score; CI, confidence interval; CV, cardiovascular; HR, hazard ratio.

**Table 3:** Cox regression analysis of AACS for the secondary outcomes.

	AACS	Events, *n* (%)	Model 1		Model 2		Model 3		Model 4	
			HR (95% CIs)	*p* Value	HR (95% CIs)	*p* Value	HR (95% CIs)	*p* Value	HR (95% CIs)	*p* Value
Fatal and non-fatal CV event	0	72 (5.3)	Reference		Reference		Reference		Reference	
	1–2	39 (11.2)	2.149 (1.384, 3.337)	<0.001	1.555 (1.036, 2.333)	0.033	1.357 (0.875, 2.103)	0.176	1.372 (0.867, 2.169)	0.177
	3–4	20 (12.0)	2.129 (1.213, 3.738)	0.009	1.246 (0.741, 2.095)	0.408	1.087 (0.615, 1.922)	0.773	1.114 (0.620, 2.001)	0.719
	5–6	12 (13.0)	2.440 (1.214, 4.906)	0.012	1.123 (0.587, 2.147)	0.725	0.873 (0.419, 1.819)	0.717	0.950 (0.447, 2.018)	0.893
	≥7	24 (19.7)	4.941 (3.039,8.035)	<0.001	1.733 (1.037, 2.896)	0.036	1.806 (1.061, 3.075)	0.029	1.052 (1.030, 1.073)	<0.001
All-cause death	0	57 (4.2)	Reference		Reference		Reference		Reference	
	1–2	19 (5.5)	1.512 (0.863, 2.648)	0.149	0.790 (0.460, 1.357)	0.393	0.727 (0.403, 1.311)	0.290	0.746 (0.413, 1.346)	0.330
	3–4	15 (9.0)	1.749 (0.889, 3.441)	0.106	0.955 (0.522, 1.747)	0.882	0.763 (0.388, 1.503)	0.435	0.751 (0.370, 1.523)	0.427
	5–6	14 (15.2)	4.011 (2.139,7.522)	< 0.001	1.272 (0.670, 2.416)	0.462	1.076 (0.533, 2.171)	0.839	1.219 (0.595, 2.499)	0.589
	≥7	24 (19.7)	5.600 (3.338, 9.395)	< 0.001	1.703 (1.005, 2.885)	0.048	1.826 (1.039, 3.208)	0.036	1.949 (1.073, 3.539)	0.028

Model 1, unadjusted model. Model 2, model 1 + adjusted for age, sex, Charlson comorbidity index, primary renal disease, current smoking status, medication (ACEi/ARBs, diuretics, number of anti-HTN drugs, statins), BMI, and SBP. Model 3, model 2 + adjusted for hemoglobin, albumin, fasting glucose, HDL-C, TG, 25(OH) vitamin D, hs-CRP, GFR and spot urine ACR. Model 4, model 3 + adjusted for LVEF and categorized E/e’ at the baseline. Abbreviations: AACS, abdominal aortic calcification score; CI, confidence interval; CV, cardiovascular; HR, hazard ratio.

**Table 4:** Cox regression analysis of AACS for the primary outcome (non-fatal CV events and all-cause death) in various subgroups.

	AACS	Events, *n* (%)	Unadjusted HR (95% CIs)	*p* for Interaction	Adjusted HR (95% CIs)	*p* for Interaction
Age < 60 years	0	64 (6.2)	Reference	0.495	Reference	0.859
	1–2	20 (10.0)	1.880 (1.115, 3.169)		1.063 (0.570, 1.982)	
	3–4	11 (14.3)	2.336 (1.223, 4.461)		0.909 (0.408, 2.023)	
	5–6	2 (8.3)	1.416 (0.345, 5.804)		0.880 (0.203, 3.819)	
	≥7	5 (21.7)	3.334 (1.333, 8.336)		0.875 (0.279, 2.741)	
Age ≥ 60 years	0	55 (17.2)	Reference		Reference	
	1–2	34 (23.0)	1.538 (0.997, 2.372)		1.125 (0.672, 1.883)	
	3–4	16 (17.8)	1.070 (0.612, 1.869)		0.828 (0.427, 1.604)	
	5–6	20 (29.4)	1.864 (1.105, 3.144)		1.175 (0.637, 2.167)	
	≥7	35 (35.4)	2.554 (1.662, 3.926)		2.038 (1.211, 3.428)	
Male	0	81 (10.2)	Reference	0.067	Reference	0.206
	1–2	39 (17.3)	1.855 (1.250, 2.753)		1.122 (0.702, 1.791)	
	3–4	25 (22.5)	2.400 (1.525, 3.775)		1.202 (0.699, 2.067)	
	5–6	18 (29.5)	3.167 (1.869, 5.365)		1.232 (0.665, 2.281)	
	≥7	30 (37.0)	4.320 (2.825, 6.608)		2.024 (1.211, 3.383)	
Female	0	38 (6.8)	Reference		Reference	
	1–2	15 (12.3)	2.167 (1.181, 3.976)		1.399 (0.678, 2.885)	
	3–4	2 (3.6)	0.427 (0.103, 1.776)		0.386 (0.088, 1.699)	
	5–6	4 (12.9)	1.733 (0.616, 4.787)		0.849 (0.230, 3.134)	
	≥7	10 (24.4)	3.450 (1.656, 7.186)		1.930 (0.698, 5.335)	
BMI < 23 kg/m^2^	0	40 (8.7)	Reference	0.243	Reference	0.175
	1–2	12 (11.4)	1.330 (0.693, 2.553)		0.525 (0.219, 1.262)	
	3–4	10 (23.3)	2.516 (1.249, 5.066)		1.051 (0.399, 2.768)	
	5–6	5 (23.8)	2.431 (0.954, 6.192)		0.698 (0.224, 2.176)	
	≥7	8 (25.8)	2.771 (1.231, 6.237)		0.834 (0.292, 2.381)	
BMI ≥ 23 kg/m^2^	0	79 (8.9)	Reference		Reference	
	1–2	42 (17.5)	2.323 (1.578, 3.420)		1.350 (0.859, 2.123)	
	3–4	16 (13.1)	1.473 (0.857, 2.530)		0.758 (0.401, 1.434)	
	5–6	17 (24.3)	2.928 (1.703, 5.035)		1.286 (0.677, 2.445)	
	≥7	32 (35.2)	4.670 (3.078, 7.084)		2.647 (1.590, 4.404)	
eGFR ≥ 45 mL/min/1.73 m^2^	0	44 (5.8)	Reference	0.022	Reference	0.206
	1–2	24 (16.2)	3.286 (1.924, 5.614)		1.635 (0.812, 3.296)	
	3–4	7 (13.0)	2.124 (0.943, 4.785)		1.226 (0.474, 3.170)	
	5–6	3 (10.0)	1.118 (0.269, 4.648)		0.542 (0.115, 2.553)	
	≥7	10 (35.7)	7.180 (3.445, 14.963)		2.772 (1.028, 7.475)	
eGFR < 45 mL/min/1.73 m^2^	0	75 (12.6)	Reference		Reference	
	1–2	30 (15.0)	1.423 (0.931, 2.175)		0.977 (0.601, 1.588)	
	3–4	30 (17.7)	1.565 (0.955, 2.564)		0.720 (0.393, 1.320)	
	5–6	19 (30.6)	3.010 (1.818, 4.984)		1.201 (0.634, 2.277)	
	≥7	30 (31.9)	3.190 (2.085, 4.880)		1.504 (0.880, 2.571)	
Spot urine ACR < 300 mg/gCr	0	62 (9.6)	Reference	0.131	Reference	0.305
	1–2	20 (13.0)	1.521 (0.911, 2.538)		0.814 (0.417, 1.588)	
	3–4	9 (12.9)	1.280 (0.632, 2.589)		0.695 (0.307, 1.575)	
	5–6	6 (18.8)	1.350 (0.541, 3.369)		0.616 (0.218, 1.746)	
	≥7	11 (26.2)	2.771 (1.450, 5.297)		1.280 (0.551, 2.972)	
Spot urine ACR ≥ 300 mg/gCr	0	55 (8.2)	Reference		Reference	
	1–2	33 (17.6)	2.433 (1.554, 3.811)		1.586 (0.960, 2.619)	
	3–4	16 (18.0)	2.218 (1.264, 3.890)		1.134 (0.585, 2.198)	
	5–6	15 (26.8)	4.011 (2.253, 7.141)		1.725 (0.890, 3.342)	
	≥7	29 (38.2)	5.664 (3.567, 8.996)		2.829 (1.596, 5.014)	

The model was adjusted for age, sex, Charlson comorbidity index, primary renal disease, current smoking status, medication (ACEi/ARBs, diuretics, number of anti-HTN drugs, statins), BMI, SBP, hemoglobin, albumin, fasting glucose, HDL-C, TG, 25(OH) vitamin D, hs-CRP, eGFR, spot urine ACR, LVEF, and categorized E/e’ at the baseline. Abbreviations: AACS, abdominal aortic calcification score; ACR, albumin-to-creatinine ratio; CI, confidence interval; Cr, creatinine; eGFR, estimated glomerular filtration rate; HR, hazard ratio.

## Data Availability

The raw data supporting the conclusions of this article will be made available by the authors, without undue reservation.

## Supplementary Materials

The following supporting information can be downloaded at: https://www.mdpi.com/article/10.3390/jcm11051157/s1, Figure S1: Kaplan–Meier curve for cumulative incidence of fatal and non-fatal CV events by AACS; Figure S2: Kaplan–Meier curve for cumulative incidence of all-cause death by AACS; Table S1: Summary of echocardiographic findings of study participants by AACS; Table S2: Cox regression analysis of AACS for composite CV events in the subjects excluding CKD stage 5; Table S3: Cox regression analysis of AACS for composite CV events in the subjects excluding CKD stage 1; Table S4: Cox regression analysis of AACS for composite CV events using a multiple imputation.
