# Supplementary material for: Abdominal Aortic Calcification and Cardiovascular Outcomes in Chronic Kidney Disease: Findings from KNOW-CKD Study
Source: J Clin Med. 2022 Feb 22;11(5):1157. doi: 10.3390/jcm11051157 (PMC8911161; doi:10.3390/jcm11051157)
Supplement: Supplementary file 1 [file jcm-11-01157-s001.zip › jcm-1574836-supplementary.pdf]

# - Supplementary Materials -

Abdominal aortic calcification and cardiovascular outcomes in chronic kidney disease: findings from KNOW-CKD study

Sang Heon Suh, M.D., Ph.D.[1], Tae Ryom Oh, M.D., Ph.D.[1], Hong Sang Choi, M.D., Ph.D.[1], Chang Seong Kim, M.D., Ph.D.[1], Eun Hui Bae, M.D., Ph.D.[1], Kook-Hwan Oh, M.D., Ph.D.[2], Joongyub Lee, M.D., Ph.D.[3], Yun Kyu Oh, M.D., Ph.D.[4], Ji Yong Jung, M.D., Ph.D[5], Seong Kwon Ma*, M.D., Ph.D.[1], and Soo Wan Kim*, M.D., Ph.D.[1], on behalf of the Korean Cohort Study for Outcomes in Patients With Chronic Kidney Disease (KNOW-CKD) Investigators

[1]Department of Internal Medicine, Chonnam National University Medical School and Chonnam National University Hospital, Gwangju, Korea

[2]Department of Internal Medicine, Seoul National University Hospital, Seoul, Korea

[3]Department of Prevention and Management, School of Medicine, Inha University, Incheon, Korea

[4]Department of Internal Medicine, Seoul National University Boramae Medical Center, Seoul, Korea

[5]Department of Internal Medicine, Division of Nephrology, Gachon University of Gil Medical Center, Incheon, Korea

Corresponding authors

*Seong Kwon Ma, M.D., Ph.D., Department of Internal Medicine, Chonnam National University Medical School, 42 Jebongro, Gwangju 61469, Korea, Tel: +82-62-220-6579, Fax: +82-62-225-8578, Email: drmsk@hanmail.net

*Soo Wan Kim, M.D., Ph.D., Department of Internal Medicine, Chonnam National University Medical School, 42 Jebongro, Gwangju 61469, Korea, Tel: +82-62-225-6271, Fax: +82-62-220-8578, Email: skimw@chonnam.ac.kr

Table of Contents

Supplemental Figure S1. Kaplan-Meier curve for cumulative incidence of fatal and non-fatal CV events by AACS

Supplemental Figure S2. Kaplan-Meier curve for cumulative incidence of all-cause death by AACS

Supplemental Table S1. Summary of echocardiographic findings of study participants by AACS

Supplemental Table S2. Cox regression analysis of AACS for composite CV events in the subjects excluding CKD stage 5

Supplemental Table S3. Cox regression analysis of AACS for composite CV events in the subjects excluding CKD stage 1

Supplemental Table S4. Cox regression analysis of AACS for composite CV events using a multiple imputation

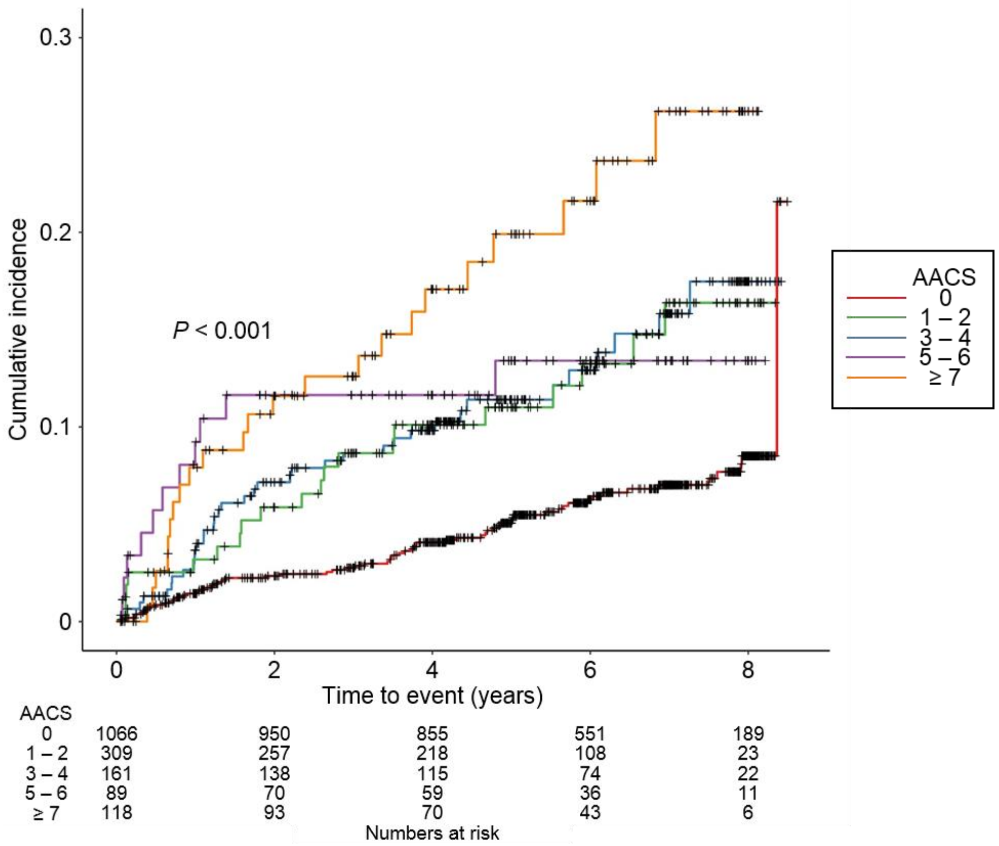

Supplemental Figure S1. Kaplan-Meier curve for cumulative incidence of fatal and non-fatal CV events by AACS

Note: *P* value by Log-rank test. Abbreviations: AACS, abdominal aortic calcification score; CV, cardiovascular.

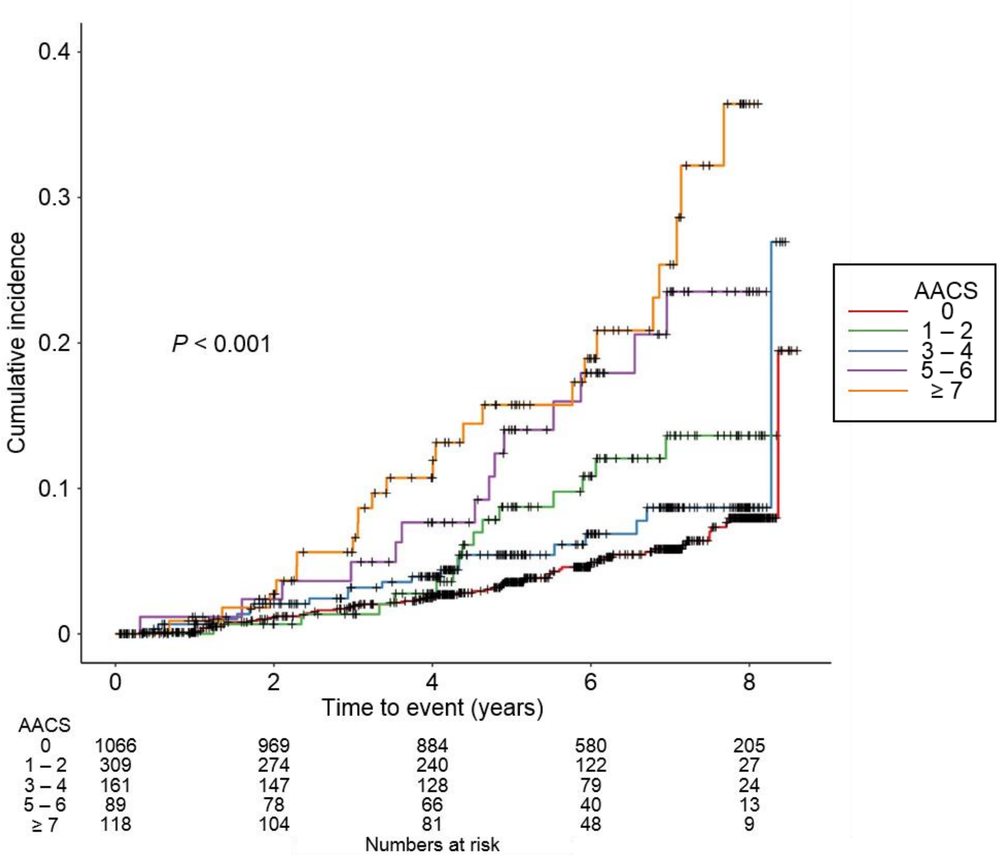

Supplemental Figure S2. Kaplan-Meier curve for cumulative incidence of all-cause death by AACS

Note: *P* value by Log-rank test. Abbreviations: AACS, abdominal aortic calcification score; CV, cardiovascular.

Supplemental Table S1. Summary of echocardiographic findings of study participants by AACS

	AACS					
	0	1 – 2	3 – 4	5 – 6	≥ 7	*P* value
LVEF (%)	64.097 ± 6.122	64.249 ± 6.368	64.087 ± 6.711	63.359 ± 7.142	64.005 ± 6.903	0.882
E/e’	9.307 ± 3.330	10.630 ± 4.145	11.316 ± 3.747	12.286 ± 7.332	11.758 ± 3.522	< 0.001
E/e’ > 14	112 (8.4)	55 (16.4)	33 (20.1)	20 (22.0)	21 (18.1)	< 0.001
RWMA	25 (1.9)	14 (4.0)	7 (4.2)	8 (8.9)	8 (6.6)	0.001
Valve Calcification	70 (5.2)	36 (10.4)	28 (16.9)	20 (22.2)	26 (21.5)	< 0.001
LVMI (g/m^2)	90.800 ± 24.446	96.337 ± 24.661	102.079 ± 25.449	99.978 ± 25.040	105.162 ± 26.596	< 0.001
PWT (mm)	9.044 ± 1.540	9.584 ± 1.614	9.584 ± 1.556	9.734 ± 1.601	10.039 ± 1.596	< 0.001
IVWT (mm)	9.111 ± 1.658	9.630 ± 1.684	9.743 ± 1.746	9.968 ± 1.675	10.274 ± 1.981	< 0.001
LVEDD (mm)	48.624 ± 4.404	48.612 ± 4.733	49.811 ± 4.743	48.810 ± 4.843	48.646 ± 4.240	0.050
LVESD (mm)	30.366 ± 3.959	30.310 ± 4.915	31.263 ± 5.028	31.079 ± 5.137	30.257 ± 3.910	0.157

Note: Values for categorical variables are given as number (percentage); values for continuous variables, as mean ± standard deviation or median [interquartile range]. Abbreviations: AACS, abdominal aortic calcification score; E/e’, ratio of the early transmitral blood flow velocity to early diastolic velocity of the mitral annulus; IVWT, interventricular wall thickness; LVEDD, left ventricular end-diastolic diameter; LVEF, left ventricular ejection fraction; LVESD, left ventricular end-systolic diameter; LVMI, left ventricular mass index; PWT, posterior wall thickness; RMWA, regional wall motion abnormality.

Supplemental Table S2. Cox regression analysis of AACS for composite CV events in the subjects excluding CKD stage 5

	AACS	Events, n (%)	Model 1		Model 2		Model 3		Model 4	
			HR (95%CIs)	*P* value	HR (95%CIs)	*P* value	HR (95%CIs)	*P* value	HR (95%CIs)	*P* value
Composite CV events	0	109 (8.5)	Reference		Reference		Reference		Reference	
	1 – 2	51 (15.8)	1.982 (1.367, 2.875)	< 0.001	1.262 (0.890, 1.791)	0.192	1.090 (0.747, 1.592)	0.654	1.139 (0.771, 1.681)	0.514
	3 – 4	26 (16.9)	1.828 (1.119, 2.988)	0.016	1.004 (0.639, 1.579)	0.985	0.899 (0.546, 1.481)	0.677	0.939 (0.564, 1.564)	0.809
	5 – 6	18 (20.9)	2.625 (1.524, 4.521)	< 0.001	0.991 (0.580, 1.691)	0.972	0.852 (0.479, 1.516)	0.586	0.904 (0.502, 1.628)	0.738
	≥ 7	35 (30.7)	4.580 (3.056, 6.865)	< 0.001	1.584 (1.035, 2.426)	0.034	1.582 (1.012, 2.473)	0.044	1.837 (1.155, 2.921)	0.010

Note: Model 1, unadjusted model. Model 2, model 1 + adjusted for age, sex, Charlson comorbidity index, primary renal disease, Current smoking status, medication (ACEi/ARBs, diuretics, number of anti-HTN drugs, statins), BMI, and SBP. Model 3, model 2 + adjusted for hemoglobin, albumin, fasting glucose, HDL-C, TG, 25(OH) vitamin D, hs-CRP, GFR and spot urine ACR. Model 4, model 3 + adjusted for LVEF and categorized E/e’ at the baseline.

Abbreviations: AACS, abdominal aortic calcification score; CI, confidence interval; CV, cardiovascular; HR, hazard ratio.

Supplemental Table S3. Cox regression analysis of AACS for composite CV events in the subjects excluding CKD stage 1

	AACS	Events, n (%)	Model 1		Model 2		Model 3		Model 4	
			HR (95%CIs)	*P* value	HR (95%CIs)	*P* value	HR (95%CIs)	*P* value	HR (95%CIs)	*P* value
Composite CV events	0	111 (10.4)	Reference		Reference		Reference		Reference	
	1 – 2	52 (16.8)	1.694 (1.174, 2.446)	0.005	1.255 (0.891, 1.767)	0.193	1.103 (0.761, 1.599)	0.604	1.114 (0.760, 1.633)	0.581
	3 – 4	27 (16.8)	1.458 (0.902, 2.357)	0.124	0.973 (0.625, 1.514)	0.904	0.837 (0.515, 1.359)	0.472	0.855 (0.521, 1.403)	0.535
	5 – 6	21 (23.6)	2.571 (1.556, 4.249)	< 0.001	1.162 (0.708, 1.907)	0.552	1.038 (0.611, 1.764)	0.890	1.138 (0.663, 1.953)	0.639
	≥ 7	39 (33.1)	4.024 (2.724, 5.945)	< 0.001	1.596 (1.066, 2.390)	0.023	1.693 (1.106, 2.593)	0.015	1.872 (1.201, 2.919)	0.006

Note: Model 1, unadjusted model. Model 2, model 1 + adjusted for age, sex, Charlson comorbidity index, primary renal disease, Current smoking status, medication (ACEi/ARBs, diuretics, number of anti-HTN drugs, statins), BMI, and SBP. Model 3, model 2 + adjusted for hemoglobin, albumin, fasting glucose, HDL-C, TG, 25(OH) vitamin D, hs-CRP, GFR and spot urine ACR. Model 4, model 3 + adjusted for LVEF and categorized E/e’ at the baseline.

Abbreviations: AACS, abdominal aortic calcification score; CI, confidence interval; CV, cardiovascular; HR, hazard ratio.

Supplemental Table S4. Cox regression analysis of AACS for composite CV events using a multiple imputation

	AACS	Model 1		Model 2		Model 3		Model 4	
		HR (95%CIs)	*P* value	HR (95%CIs)	*P* value	HR (95%CIs)	*P* value	HR (95%CIs)	*P* value
Composite CV events	0	Reference		Reference		Reference		Reference	
	1 – 2	2.222 (1.608, 3.070)	< 0.001	1.278 (0.914, 1.787)	0.152	1.306 (0.933, 1.828)	0.121	1.317 (0.941, 1.844)	0.110
	3 – 4	2.091 (1.376, 3.177)	< 0.001	1.016 (0.658, 1.568)	0.944	0.971 (0.627, 1.504)	0.895	0.990 (0.639, 1.533)	0.962
	5 – 6	3.352 (2.125, 5.287)	< 0.001	1.197 (90.736, 1.947)	0.469	1.193 (0.734, 1.940)	0.477	1.227 (0.754, 1.998)	0.410
	≥ 7	4.991 (3.482, 7.154)	< 0.001	1.665 (1.117, 2.481)	0.013	1.641 (1.094, 2.463)	0.018	1.604 (1.062, 2.422)	0.026

Note: Model 1, unadjusted model. Model 2, model 1 + adjusted for age, sex, Charlson comorbidity index, primary renal disease, Current smoking status, medication (ACEi/ARBs, diuretics, number of anti-HTN drugs, statins), BMI, and SBP. Model 3, model 2 + adjusted for hemoglobin, albumin, fasting glucose, HDL-C, TG, 25(OH) vitamin D, hs-CRP, GFR and spot urine ACR. Model 4, model 3 + adjusted for LVEF and categorized E/e' at the baseline.

Abbreviations: AACS, abdominal aortic calcification score; CI, confidence interval; CV, cardiovascular; HR, hazard ratio.
